# Predictors of health care use by adults 50 years and over in a rural South African setting

**DOI:** 10.3402/gha.v7.24771

**Published:** 2014-08-01

**Authors:** Soter Ameh, Francesc Xavier Gómez-Olivé, Kathleen Kahn, Stephen M. Tollman, Kerstin Klipstein-Grobusch

**Affiliations:** 1 MRC/Wits Rural Public Health and Health Transitions Research Unit (Agincourt), School of Public Health, Faculty of Health Sciences, University of the Witwatersrand, Johannesburg, South Africa; 2 Community Medicine Department, College of Medical Sciences, University of Calabar, Cross River State, Nigeria; 3 INDEPTH Network, Accra, Ghana; 4 Umeå Centre for Global Health Research, Epidemiology and Global Health, Umeå University, Umeå, Sweden; 5 Division of Epidemiology and Biostatistics, School of Public Health, University of the Witwatersrand, Johannesburg, South Africa; 6 Julius Global Health, Julius Centre for Health Sciences and Primary Care, University Medical Centre Utrecht, Utrecht, The Netherlands

**Keywords:** older adults, chronic diseases, predictors, health care use, Agincourt, South Africa

## Abstract

**Background:**

South Africa’s epidemiological transition is characterised by an increasing burden of chronic communicable and non-communicable diseases. However, little is known about predictors of health care use (HCU) for the prevention and control of chronic diseases among older adults.

**Objective:**

To describe reported health problems and determine predictors of HCU by adults aged 50+ living in a rural sub-district of South Africa.

**Design:**

A cross-sectional study to measure HCU was conducted in 2010 in the Agincourt sub-district of Mpumalanga Province, an area underpinned by a robust health and demographic surveillance system. HCU, socio-demographic variables, reception of social grants, and type of medical aid were measured, and compared between responders who used health care services with those who did not. Predictors of HCU were determined by binary logistic regression adjusted for socio-demographic variables.

**Results:**

Seventy-five percent of the eligible adults aged 50+ responded to the survey. Average age of the targeted 7,870 older adults was 66 years (95% CI: 65.3, 65.8), and there were more women than men (70% vs. 30%, *p*<0.001). All 5,795 responders reported health problems, of which 96% used health care, predominantly at public health facilities (82%). Reported health problems were: chronic non-communicable diseases (41% – e.g. hypertension), acute conditions (27% – e.g. flu and fever), other conditions (26% – e.g. musculoskeletal pain), chronic communicable diseases (3% – e.g. HIV and TB), and injuries (3%). In multivariate logistic regression, responders with chronic communicable disease (OR=5.91, 95% CI: 1.44, 24.32) and non-communicable disease (OR=2.85, 95% CI: 1.96, 4.14) had significantly higher odds of using health care compared with those with acute conditions. Responders with six or more years of education had a two-fold increased odds of using health care (OR=2.49, 95% CI: 1.27, 4.86) compared with those with no formal education.

**Conclusion:**

Chronic communicable and non-communicable diseases were the most prevalent and main predictors of HCU in this population, suggesting prioritisation of public health care services for chronic diseases among older people in this rural setting.

Population ageing has been described by the United Nations as one of the most distinctive demographic events of the 20th century, and an important population challenge in the 21st century. There were, respectively, 200 and 600 million older people (60 years and over) in 1950 and 2000, and it has been projected that there will be two billion older people globally in 2050. This depicts tripling of the number of older people in two consecutive 50 years, particularly in lower- and middle-income countries (LMIC) that already hosted 62% of the world’s older population in 2000 (1).

In Africa, older people constituted 5.1% of the total population at the beginning of the 20th century. With the exception of Réunion (9.9%) and Mauritius (9%), the population of older people in South Africa (7.3%) was higher than the 5.1% for the African continent at the turn of the 20th century (2). It has also been projected that the population of older people in South Africa will be more than one person in 10 by 2025 (3), due to ageing and scale-up of antiretroviral drugs (ARVs).

Although there is no United Nations (UN) standard numerical criterion for old age, the agreed cut-off for defining old age is generally 60 years and above (4). While the ageing process is a biological activity, it is also a construct that is dependent on how each society makes sense of it. The chronological age of 60 or 65 is said to be the beginning of old age in most developed countries because it is roughly equivalent to retirement age (5). In Africa, the majority of older people do not expect formal retirement or retirement benefits because they live in rural communities and earn a living outside the formal sector (4). Southern Africa is the region with the highest prevalence of HIV/AIDS in the African continent. Life expectancy has decreased from 61 years in 1990–1995 to 52 years in 2005–2010 in this region due to the impact of HIV/AIDS, and is it not expected to recover from the pre-1990 levels until 2045 (6).

The Minimum Data Set (MDS) project on Ageing – supported by the World Health Organization (WHO) and the U.S. National Institute on Ageing – has set 50 years and above as the cut-off to refer to the older population in Africa (4, 7). The same cut-off has been used in the Study on Global Ageing and Adult Health (SAGE) (8). Given this evidence and the fact that the global population of persons aged 50 and over is expected to increase from 21% to 30% in 2050 (6), this paper adopts 50 years and above to describe the population of older adults.

One of the expected consequences of ageing is increasing prevalence of non-communicable diseases (NCDs). In 2008, NCDs were responsible for two-thirds of all mortality worldwide – with 80% of these deaths occurring in LMICs (9). But quite unlike children and women whose health problems have been included in the agenda of the Millennium Development Goals (MDGs), those of older people were not clearly visible in most global policy dialogue until 2011 (10). This could have implications for health care services for older adults in LMICs (2), particularly if health systems are ill-prepared to deal with the long-term care for the management of chronic diseases (11).

The Constitution of the Republic of South Africa asserts that ‘everyone has the right to have access to health care services’ (12). Successive post-apartheid African National Congress-led governments have continued to consolidate on the pro-equity policies, many of which are elements of the Reconstruction and Development Programme (RDP). The health-related component of the RDP includes free Primary Health Care (PHC) for every person using public health facilities and waived income-related user fees in public hospitals (13). Consequently, there has been an improvement in the use of public PHC services by all age groups in South Africa (14, 15). Increasing use of health services has also been reported in Uganda and Mali, where cost sharing or user fees were discontinued (16, 17).

With the increasing prevalence of NCDs in South Africa mainly due to ageing, lifestyle changes and expanded ARV roll-out (11), increase in health care demands are anticipated. There is a plethora of evidence of increasing health problems (8, 11, 18–21) and self-reported health care use (HCU) among older people (18, 21). Yet, the predictors of HCU to tailor provision of services for the prevention and control of chronic diseases have not been addressed in detail. The purpose of this study was to describe the health problems of adults 50 years and over living in a rural South African population, and to determine the predictors of HCU.

## Methodology

### Study setting

The study used data from the MRC/Wits Agincourt Research Unit situated in Ehlanzeni Health District, Mpumalanga Province, South Africa. Trained local field workers collect and update vital events (births, deaths, and migration) on a yearly basis since 1992. This is complemented by additional information at different time intervals such as on HCU, education level, labour, migration, and household assets (22). The current population under follow-up in the MRC/Wits Agincourt Research study site as on 1^st^ July 2011 was approximately 90,000 people in 16,000 households living in 27 villages (23).

The study area covers about 420 km^2^. Tsonga is the most widely spoken language. One-third of the population are of Mozambican origin, having immigrated into South Africa mainly as war refugees in the early- and mid-1980s. Despite the current government’s development initiatives, which have led to improved housing, access to potable water, electricity and social security grants, infrastructure in the area is still limited. Unemployment rates remains high, with 60% of labour migration being accounted for by men aged 35–54 years and an increasing proportion of labour migrants seen among young men and women (22). The pattern of labour migration has resulted in a disproportionately higher proportion of older women permanently resident in the area. There are eight health facilities in the study area: one public health centre, six government satellite clinics, and one private community health centre in a public–private partnership. There are three referral hospitals situated 25 and 45 km from the study setting (23).

### Health service infrastructure

The government of South Africa decentralised the provision of health services by dividing the country into 53 health districts to ensure that citizens have access to a comprehensive package of PHC and district hospital services. In the South African PHC model, the nurse is the provider of services in the clinics and comprehensive health centres – which are the first point of entry to the health system. Located within the reach of rural, semi-rural, and urban communities, these facilities are the cornerstone of the public health system through provision of comprehensive and integrated ‘preventive, promotional, curative and rehabilitation services’ (24).

The range of services includes maternal and childcare, immunisation, family planning, treatment of sexually transmitted infections, minor trauma, and care for chronic diseases (e.g. diabetes and hypertension). Additional services provided by the health centres include 24-hour maternity services, accident and emergency services, up to 30 beds for observation for a maximum of 48 hours, a procedure room (not an operating theatre). With the exception of emergency cases which are referred to the hospitals (secondary level of care), the clinics and health centres offer services to ambulatory patients for 8 hours/day and 24 hours, respectively (24).

### Study design and sampling

**Fig. 1:**
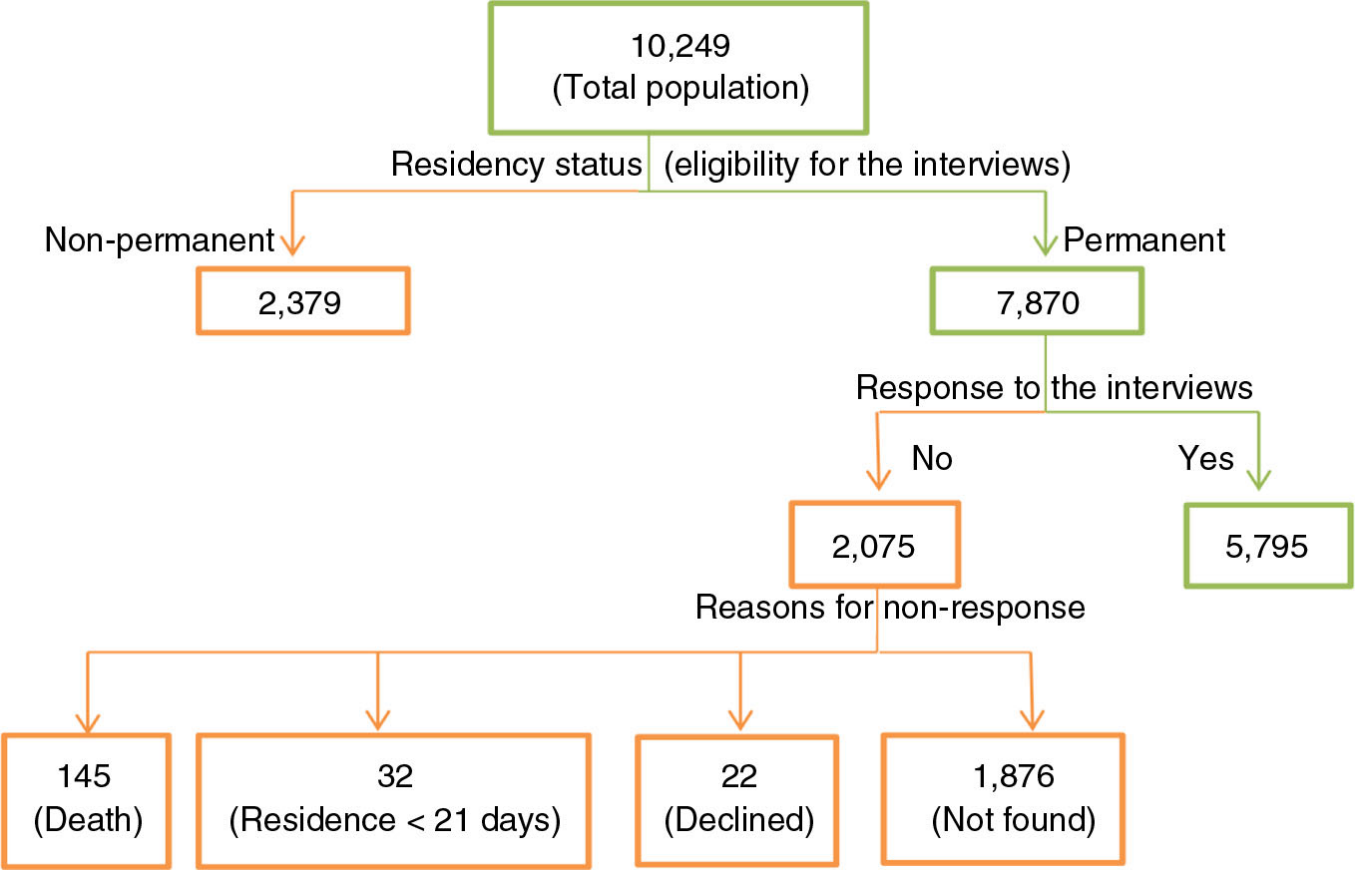
Sampling of eligible study participants.

This was a cross-sectional study of all eligible older adults aged 50 years and older in the study site. Of the total 10,249 older adults registered in the 2009 census database, 7,870 persons with permanent residency status were eligible and targeted for the 2010 HCU survey. Eligibility criteria for the interviews were 1) residency status of 21 days or more before the survey for those prospective participants who moved out of the study site after the 2009 census and relocated to the study site before the 2010 survey and 2) availability of the prospective participants at home after two revisits by field workers (Fig. 1).

### Training and quality control

Field workers were trained for two days in the administration of the HCU questionnaire, as part of preparation for the general census. Field work was closely supervised for a week, after which a new training session was run to review and tackle challenges. Quality control followed a four-step system where field workers, supervisors, quality checkers, and data entry clerks assured good quality of the data: 1) The field workers double-checked all questionnaires before leaving the household of the interviewee, and again at the office before submission to the supervisors. 2) The supervisors randomly checked the questionnaires for inconsistencies and blank questions before submitting to the quality checkers. 3) The quality checkers identified inconsistencies and other errors in the questionnaires before submitting to the data entry clerks. 4) Data entry clerks identified forms with errors during data entry and returned them to the field for correction, after which the whole process of quality control was engaged prior to final data entry.

The questionnaire for the 2010 adult HCU survey was based on a HCU questionnaire used previously in the site to gather information on the older adult population (18). The questionnaire was used to collect information on socio-demographic variables, reception of any type of social grant, access to medical aid, need for and access to health care, type of disease, disability and hospitalisation.

### Variables

Age in years was calculated on 1^st^ August 2010 using the census date of birth for all potential participants. Responders were then categorised into 10-year age intervals: 50–59, 60–69, and 70+. Years of formal education were obtained from the 2007 MRC/Wits Agincourt Research Unit database, which was the latest updated information. Years of education were categorised according to the WHO levels of education: no formal education, <6 years, and ≥6 years. Medical aid was categorised to reflect responders with: 1) medical aid to visit the doctor, 2) health insurance for specific disease, 3) medical aid in employer’s clinic/hospital, 4) access to free public hospital care, and 5) no medical aid/do not know. The variable ‘last time health care was needed’ was categorised into: 1) <1 year, 2) 1–3 years, 3) >3 years, and 4) never. In order to minimise errors due to recall bias, analysis of the predictors of HCU was restricted to responders who reported needing health care less than one year preceding the survey. The justification for using less than one year as the cut-off was based on the assumption that it is easier for responders to recall experiences with HCU in <1 year than in 1–3 years or >3 years.

Due to the influx of Mozambican refugees into Agincourt sub-district, nationality of origin was grouped into South Africans and Mozambicans. Socio-economic status (SES) was constructed from a household asset score in the 2009 census data. A principal component factor analysis technique was used to construct SES based on 30 variables on access to water and electricity, type and size of dwelling, appliances, ownership of livestock, and transport available. Subsequently, responders were categorised into quintiles in the ascending order of lowest, middle low, middle, middle high, and highest SES (25).

In order to ascertain employment status, the variable ‘looking for a paid job’ was categorised as yes or no. Reception of social grant was recorded as none, old age and disability. Hospitalisation, HCU (defined as the need for and access to health care, at least once, less than one year before the HCU survey in 2010) and disability were all binary variables (yes vs. no). Apart from acute conditions (fever and flu) and chronic communicable disease (HIV and TB), other types of illness were generated by recoding the reasons for visiting health facilities into NCD (hypertension, diabetes, stroke, sleep disorder, chronic pain in joints, depression, anxiety, cancer, and heart problems), injuries and others type of illness (musculoskeletal pain and nutritional deficiency). Actions taken during an illness episode less than one year before the survey were: visiting public and private health facilities. Other actions included practicing self-medication, consulting faith/traditional healers, and taking no action.

### Statistical analysis

Validation checks were done during data entry in MicrosoftSQL server 2005 database. Data were extracted into Stata 12.0 (College Station, TX, USA) for statistical analysis. At *p*-value of 0.05, bivariate analysis compared responders who used health care services with those who did not. The cut-off point for univariate binary logistic regression analysis was set at *p*-value of ≤0.2 and variables that were significantly associated with HCU were used to model the multivariate binary logistic regression analysis (*p*-value ≤0.05). Multiple imputation by chained equations (MICE) approach for categorical variables was used to impute for ‘socio-economic status’ and ‘looking for a paid job’, which had 1.3% and 12.4% missing values, respectively. Multiple imputation is a simulation-based method for analysing incomplete variables. It predicts missing values as close as possible to the true ones by replacing missing data with probable values based on other available information (26). Imputation is considered to have less estimation bias and valid statistical inference than list-wise deletion because the latter leads to loss of statistical power (27).

### Ethical clearance

Ethical clearance for the MRC/Wits Rural Public Health and Health Transitions Research Unit (Agincourt) was granted by the Committee for Research on Human Subjects (Medical) of the University of the Witwatersrand, Johannesburg, South Africa (Ref No. M960720).

## Results

### Comparison of responders with non-responders

Of the 10,249 adults aged 50 years and older registered in the rosters of the 2009 census, analysis was restricted to 7,870 (77%) older adults who were permanently resident in the study site. Of those, 5,795 responded to the HCU questionnaire while 32 people (0.4%) were ineligible because they lived in the study site less than 21 days before the survey. We were unable to contact 1,876 people (23.8%) who could not be found at home by the field worker after two revisits. Others who could not participate in the survey were 145 (1.9%) who died before the survey and 22 (0.3%) who declined participation. Response rate, defined as the number of respondents divided by the number of eligible subjects in the sample (28), was 75% [5,795/(7870–145)] (Fig. 1).

A comparison of 5,795 responders with 2,075 non-responders (Appendix A) showed that the responders were older (mean age=66 vs. 64 years, *p*<0.001) and predominantly women (74.8% vs. 55.4%, *p*<0.001), whereas non-responders had more years of formal education (19.1% vs. 12.9%, *p*<0.001) and highest asset score (25.9% vs. 22.5%, *p*<0.001).

Analysis of socio-demographic characteristics, by gender, of the 5,795 adults who responded to the 2010 HCU survey showed significant differences in all variables, except nationality (data not shown). The men were older (mean age=67 years, 95% CI 66.1; 67.1) than the women (mean age=66 years, 95% CI 65.6; 66.3). The men also had more years of formal education (18% vs. 11.0%, *p*<0.001) and higher asset score (26% vs. 22%, *p*=0.011). More men than women were presently working for cash payment (17% vs. 12%, *p*<0.001), looking for a paid job (12% vs. 10%, *p*=0.019), and receiving an old age grant (61% vs. 59%, *p*<0.001).

### Reasons for using health care

Of the 5,795 respondents, 5,056 (87%) needed health care less than one year preceding the survey (Table 1). From these, 4,877 (96%) reported using health care services. The majority (82%) of the 4,877 who used health care visited public health facilities. In descending order, reasons for needing health care less than one year preceding the survey included: NCD −41%, acute conditions −27%, other conditions −26%, chronic communicable disease −3%, and injuries −3%. Of the 5,056 adults who needed health care less than one year preceding the survey, 1,344 (27%) were hospitalised, while 274 (5%) had disabilities. Table 1 also showed significant differences (*p*<0.001) in health problems by gender, but there was no significant difference by gender in disability (*p*=0.353) and HCU (*p*=0.164).

**Table 1:** Health-seeking behaviour, by gender among 5,795 responders to the adult HCU survey in Agincourt sub-district in 2010

	Women	Men	Total	
	
Variables	n (%)	n (%)	n (%)	*p*
Last time health care was needed (n=5,795)				
<1 year	3,863 (89.2)	1,193 (81.6)	5,056 (87.2)	<0.001
1–3 years	204 (4.7)	84 (5.7)	288 (5.0)	
>3 years	266 (6.1)	185 (12.7)	451 (7.8)	
Health problems needing health care <1 year before survey (n=5,056)				
Non-communicable diseases^a^	1,649 (42.7)	422 (35.4)	2,071 (41.0)	<0.001
Acute diseases^b^	1,053 (27.3)	295 (24.7)	1,348 (26.6)	
Other diseases^c^	977 (25.3)	351 (29.4)	1,328 (26.3)	
Chronic communicable disease^d^	99 (2.6)	71 (6.0)	170 (3.4)	
Injuries	85 (2.5)	54 (4.5)	139 (2.7)	
Hospitalisation <1 year before survey (n=5,056)				
No	2,866 (74.2)	846 (70.9)	3,712 (73.4)	0.025
Yes	997 (25.8)	347 (29.1)	1,344 (26.6)	
Disability requiring treatment <1 year before survey (n=5,056)				
No	3,660 (94.8)	1,122 (94.0)	4,782 (94.6)	0.353
Yes	203 (5.2)	71 (6.0)	274 (5.4)	
Health care use by responders who needed health care <1 year before survey (n=5,056)				
No	129 (3.3)	50 (4.2)	179 (3.5)	0.164
Yes	3,734 (96.7)	1,143 (95.8)	4,877 (96.5)	
Actions taken by responders who used health care <1 year before survey (n=4,877)				
Public health facility	3,132 (83.9)	881 (77.1)	4,013 (82.3)	0.001
Private health facility	239 (6.4)	120 (10.5)	359 (7.4)	
Other^e^	229 (6.1)	105 (9.2)	334 (6.9)	
Self-medication	105 (2.8)	28 (2.5)	133 (2.7)	
Faith or traditional healer	14 (0.4)	3 (0.2)	17 (0.3)	
None	15 (0.4)	6 (0.5)	21 (0.4)	

a Hypertension, diabetes, stroke, sleep disorder, chronic pain in joints, depression, anxiety, cancer, heart problems.

b Fever and flu.

c Musculoskeletal pain.

d HIV and TB

e Change diet and exercise.

### Reasons for not using health care

**Fig. 2:**
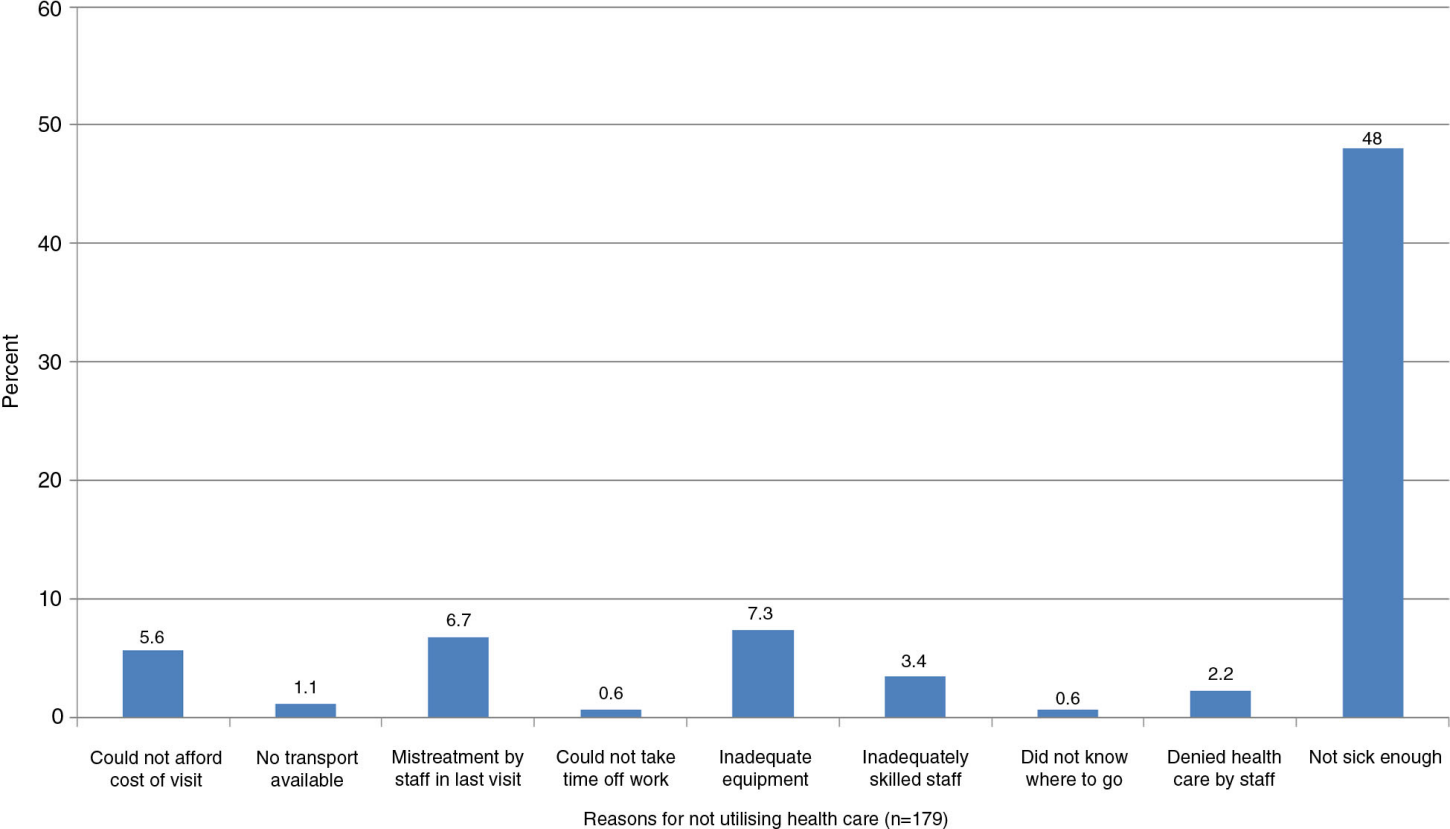
Reasons for not using health care among 179 adults who did not use health care less than one year before the survey in Agincourt sub-district, 2010. NB: Reasons for not using health care were multiple response answers.

Of the 5,056 responders who needed health care less than one year preceding the survey, 179 did not use health care for the following main reasons: 86 (48%) did not think they were sick enough, 10 (6%) could not afford the cost of health facility visit, 13 (7%) thought that drugs or treatment was seemingly inadequate, and 12 (7%) reported being treated poorly during previous visits (Fig. 2).

### Socio-demographic characteristics of adults who used public and private health facilities

Of the 4,452 adults who visited health care facilities less than one year preceding the survey, 4,089 (92%) and 363 (8%) visited public and private health care facilities, respectively (Table 2). Analysis to compare use of public and private health care facilities showed significant differences (*p*<0.05) in all socio-demographic characteristics, except age (*p*=0.093) and type of grant (*p*=0.914).

**Table 2:** Socio-demographic characteristics and type of health care facility used in Agincourt sub-district in 2010

	Type of health care facility n (%)			
	
	Public	Private	Total	
	
Variables	(n=4,089)	(n=363)	(n=4,452)	*p*
Age (years)				
50–59	1,365 (33.4)	140 (38.6)	1,505 (33.8)	0.093
60–69	1,186 (29.0)	104 (28.6)	1,290 (29.0)	
≥70	1,538 (37.6)	119 (32.8)	1,657 (37.2)	
Gender				
Female	3,186 (77.9)	243 (66.9)	3,429 (77.0)	<0.001
Male	903 (22.1)	120 (33.1)	1,023 (23.0)	
Education (completed years)				
No formal education	2,613 (63.9)	201 (55.4)	2,814 (63.2)	<0.001
≤6	979 (23.9)	73 (20.1)	1,052 (23.6)	
>6	497 (12.2)	89 (24.5)	586 (13.2)	
Nationality of origin				
South African	2,868 (70.1)	274 (75.5)	3,142 (70.6)	0.032
Mozambican	1,221 (29.9)	89 (24.5)	1,310 (29.4)	
Medical aid cover				
Free public hospital care	3,878 (94.8)	283 (77.9)	4,161 (93.5)	<0.001
Whenever I need to see the doctor	25 (0.6)	73 (20.1)	98 (2.2)	
Health insurance for specific disease	2 (0.1)	1 (0.3)	3 (0.1)	
Employer’s clinic/hospital	168 (4.1)	4 (1.1)	172 (3.8)	
No medical aid/do not know	4 (0.1)	1 (0.3)	5 (0.1)	
Occupation				
Working for cash payment	493 (12.1)	74 (20.4)	567 (12.7)	<0.001
Not working for cash payment	3,596 (87.9)	289 (79.6)	3,885 (87.3)	
Socio-economic status				
Lowest	654 (16.0)	36 (9.9)	690 (15.5)	
Middle low	837 (20.5)	58 (16.0)	895 (20.1)	
Middle	904 (22.1)	66 (18.2)	970 (21.8)	<0.001
Middle high	768 (18.8)	66 (18.2)	834 (18.7)	
Highest	870 (21.2)	137 (37.7)	1,007 (22.6)	
Missing	56 (1.4)	0 (0.0)	56 (1.3)	
Looking for a paid job				
Yes	350 (8.6)	17 (4.7)	367 (8.2)	<0.001
No	3,277 (80.1)	279 (76.8)	3,556 (79.9)	
Missing	426 (11.3)	67 (18.5)	529 (11.9)	
Type of grant				
Old age	2,415 (59.1)	211 (58.1)	2,626 (59.0)	0.914
Disability	153 (3.7)	13 (3.6)	166 (3.7)	
None	1,521 (37.2)	139 (38.3)	1,660 (37.3)	

### Predictors of HCU

Table 3 showed the results of the binary logistic regression analysis of predictors of HCU (in- and out-patient care). In the univariate binary logistic regression analysis, gender, education, nationality, looking for a paid job, and type of illness predicted HCU. In the multivariate binary logistic regression model, adults with six or more years of education had a two-fold increased odds (OR=2.13, 95% CI: 1.19, 3.82) of using health care compared to those with no formal education. Also in the multivariate model, compared with respondents with acute health conditions, those with chronic communicable diseases (OR=5.91, 95% CI: 1.44, 24.32), NCDs (OR=2.85, 95% CI: 1.96, 4.14) and other health problems (OR=1.83, 95% CI: 1.27, 2.66) had significantly higher odds of use of health care.

**Table 3:** Predictors of health care use among 5,056 adults aged 50+ who used health care less than one year before the survey in Agincourt sub-district in 2010

	Health care use (N =5,056)	
	
Variables	Univariate binary logistic regression model OR (80% CI)	Multivariate binary logistic regression model OR (95% CI)
Age (years)		
50–59	1	Not included in the final model
60–69	1.05 (0.81, 1.35)	
≥70	0.90 (0.71, 1.13)	
Gender		
Male	1	1
Female	1.27 (1.02, 1.57)	1.34 (0.96, 1.89)
Education (in completed years)		
No formal education	1	1
≤6	0.87 (0.70, 1.08)	0.85 (0.59, 1.21)
>6	2.49 (1.63, 3.81)	2.49 (1.27, 4.86)
Nationality of origin		
Mozambican	1	1
South African	1.26 (1.03, 1.55)	1.07 (0.76, 1.49)
Occupation		
Presently working for cash payment	1	Not included in the final model
Not presently working for cash payment	1.09 (0.82, 1.44)	
Socio-economic status		
Lowest	1	Not included in the final model
Middle low	0.84 (0.11, 1.17)	
Middle	0.79 (0.58, 1.09)	
Middle high	1.12 (0.79, 1.59)	
Highest	1.11 (0.79, 1.55)	
Looking for a paid job		
No	1	1
Yes	0.70 (0.49, 0.99)	0.75 (0.44, 1.28)
Type of grant		
None	1	
Old age	1.07 (0.87, 1.31)	Not included in the final model
Disability	1.63 (0.84, 3.18)	
Health problems		
Acute conditions^a^	1	1
Chronic communicable disease^b^	5.51 (2.19, 13.87)	5.91 (1.44, 24.32)
Non-communicable diseases^c^	2.95 (2.32, 3.76)	2.85 (1.96, 4.14)
Injuries	2.97 (1.39, 6.37)	3.13 (0.97, 10.08)
Other	1.83 (1.44, 2.33)	1.83 (1.27, 2.66)

a Fever and flu.

b HIV and TB.

c Hypertension, diabetes, stroke, sleep disorder, chronic pain in joints, depression, anxiety, cancer, and heart problems.

## Discussion

The main findings of this study showed a high prevalence of chronic communicable and NCDs, HCU in cases where it was needed and access to public health care facilities. The main factors predicting HCU were chronic communicable diseases and NCDs, as well as higher educational attainment.

In a setting with a high prevalence of HIV (29) and cardiovascular risk factors (30) among older people, it is expected that HCU by the study population will also increase. In the current study, almost all adults (96%) who needed health care less than one year preceding the survey used health facilities. The self-reported HCU in this study is about twice as high as previously reported for the national Study on Global Ageing and Adult Health (SAGE, 45%), which was commenced in the study site in 2006, four years earlier than this study was implemented (18).

The main health problems self-reported in the study population were (in descending order): chronic NCDs, acute diseases, other diseases, chronic communicable diseases, and injuries. Self-report of chronic communicable diseases such as HIV and TB was low (3%), and well below the 2010 national HIV prevalence (10.5%) (31), the 2005 Provincial estimate in pregnant women (32), and the local HIV prevalence among adults aged 50+ (17%) as recently estimated in the study site (29). This suggests significant under-reporting of HIV infection, possibly related to stigma associated with HIV infection (33).

The high prevalence of NCDs in the current study is a reflection of the on-going epidemiological transitional changes in South Africa, due to lifestyle changes and expanded ARV roll-out (11, 19, 20). Despite the high prevalence of NCDs, communicable diseases were a stronger predictor of HCU. This may be explained by the general low level of awareness of hypertension (34), which is the most prevalent NCD in the study site (30), possibly due to the lack of symptoms among diagnosed individuals. The main reason why 179 study participants (most of whom reported NCDs) also reported not using health care was the ‘thought of not being sick enough’. This evidence supports a study in Tanzania in which there was low use of health care services by hypertensive patients following a screening programme (34). Health education programmes that focus on continuing care through regular clinic visits could promote the health of people living with chronic diseases, who do not normally seek health care because of the thought of not being sick.

Chronic communicable diseases were the foremost predictor of HCU by responders in the study area, possibly due to the fact that those who reported being infected with HIV were already engaged in the health care system. Perhaps the reasons for which chronic communicable and NCDs predicted HCU were because of the high prevalence of these chronic conditions in the study setting (29, 30), and the fact that most of the HCU occurred in the public health facilities (the largest group used in the study setting). This may have implications for South Africa’s PHC system, which has yet to adapt to long-term continuity of care for patients living with chronic diseases (11) and those not yet accessing chronic disease services.

Similar to other studies showing education as a strong predictor of use of facility-based maternal health services (35–37), and consistent with evidence in the literature showing that higher education is an important determinant for positive behaviour or motivation towards HCU (37), this study showed that having more years of education was associated with higher HCU. In agreement with the evidence that women are more likely than men to report their blood pressure status (18, 21), female respondents in this study were observed to be more likely than their male counterparts to use health care.

The main strength of this study was the use of a community-based survey of older adults to describe self-reported health problems and health-seeking behaviours, and to determine the predictors of HCU in a rural South African setting. The main limitation of this research was information bias. This was evidently the case in the under-reporting of chronic communicable diseases, such as HIV, due to stigmatisation. The response rate was 75%. Although a response rate of at least 60% in surveys of this type is reasonable (38), non-response bias may have influenced the results. This is in view of the evidence that non-responders differed significantly in part from responders. The characteristics (relatively younger men with more years of education and highest asset score) of these non-responders have been reported among labour migrants in another research in the study site (25). There may have been recall bias among these older adult study participants in reporting their experiences with health care less than one year before the survey. Unfortunately, there was no information on the number of in- and out-patient visits and hospitalisations. The availability of these data would have enabled the authors to further interrogate and interpret the context in which the number of health facility visits or hospitalisations may have accounted for the high level of the reported HCU.

## Conclusion

Chronic communicable and NCDs were prevalent and also important predictors of HCU. This suggests the need to prioritise public health care services for chronic diseases among older people in rural South African settings. The on-going pilot implementation of the Integrated Chronic Disease Management model (39) in South Africa presents a unique opportunity to tailor health care services for the prevention and control of chronic diseases in the study area.

## Appendix

**Table:** Appendix A: Socio-demographic characteristics of responders and non-responders to the 2010 health care use survey in Agincourt sub-district

	Responders	Non-responders	Total	
Variables	(n=5,795)	(n=2,075)	(n=7,870)	*p*
Age group (years)				<0.001
50–59	1,933 (33.3)	920 (44.4)	2,853 (36.2)	
60–69	1,703 (29.4)	515 (24.8)	2,218 (28.2)	
≥70	2,158 (37.3)	640 (30.8)	2,799 (35.6)	
Mean age in years	66.1 (65.8, 66.4)	64.0 (63.5, 64.5)	65.6 (65.3, 65.8)	
Gender				<0.001
Female	4,333 (74.8)	1,149 (55.4)	5,482 (69.7)	
Male	1,462 (25.2)	926 (44.6)	2,388 (30.3)	
Education (in completed years)				<0.001
No formal education	3,680 (63.5)	1,263 (60.9)	4,943 (62.8)	
≤6	1,369 (23.6)	415 (20.0)	1,784 (22.7)	
>6	746 (12.9)	397 (19.1)	1,143 (14.5)	
Nationality of origin				0.052
South African	4,032 (69.6)	1,396 (67.3)	5,428 (69.0)	
Mozambican	1,763 (30.4)	679 (32.7)	2,442 (31.0)	
Socio-economic status				<0.001
Lowest	927 (16.0)	339 (16.4)	1,266 (16.1)	
Middle low	1,160 (20.0)	391 (18.8)	1,551 (19.7)	
Middle	1,239 (21.4)	359 (17.3)	1,598 (20.3)	
Middle high	1,091 (18.8)	421 (20.3)	1,512 (19.2)	
Highest	1,301 (22.5)	538 (25.9)	1,839 (23.4)	
Missing	77 (1.3)	27 (1.3)	104 (1.3)	
Medical aid cover				
Free public hospital care	5,422 (93.6)	–		
Whenever I need to see the doctor	118 (2.1)	–		
Health insurance for specific disease	7 (0.1)	–		
Employer’s clinic/hospital	188 (3.2)	–		
No medical aid/do not know	60 (1.0)	–		
Occupation				
Not working for cash payment	5,016 (86.6)	–		
Working for cash payment	779 (13.4)	–		
Looking for a paid job				
Yes	508 (8.8)	–		
No	4,567 (78.8)	–		
Missing	720 (12.4)	–		
Type of grant				
Old age	3,437 (59.3)	–		
Disability	182 (3.1)	–		
None	2,176 (35.6)	–		
